# A Triple Obstetric Challenge of Thoracopagus-Type Conjoined Twins, Eclampsia, and Obstructed Labor: A Case Report from Sub-Saharan Africa

**DOI:** 10.1155/2017/6815748

**Published:** 2017-12-05

**Authors:** Mariatu Binta Leigh, Valerie John-Cole, Mike Kamara, Alimamy Philip Koroma, Michael Momoh Koroma, Edward Ejiro Emuveyan, Peter Bramlage, Ivo Buschmann

**Affiliations:** ^1^Department of Obstetrics and Gynecology, Princess Christian Maternity Hospital (PCMH), University Teaching Hospitals Complex, University of Sierra Leone, Freetown, Sierra Leone; ^2^Center for Internal Medicine I, Department for Angiology, Medical School Brandenburg Theodor Fontane (MHB), Campus Brandenburg, Brandenburg, Germany; ^3^Department of Anesthesia, Princess Christian Maternity Hospital (PCMH), University Teaching Hospitals Complex, University of Sierra Leone, Freetown, Sierra Leone; ^4^Department of Obstetrics and Gynecology, College of Medicine, University of Lagos, Akoka, Lagos, Nigeria

## Abstract

Conjoined twins are very rarely seen. We present a case of thoracopagus that was undiagnosed prior to delivery and combined with eclampsia and obstructed labor in a low-resource setting in sub-Saharan Africa. A 27-year-old pregnant woman was presented to the maternity emergency unit of Princess Christian Maternity Hospital (PCMH) in Freetown at term in labor. Upon admission, the patient was awake and orientated and presented a blood pressure of 180/120 mmHg and a protein value of 3+ on urine dipstick test. Clinical examination—ultrasound was not available—led to the admission diagnosis: obstructed labor with intrauterine fetal death and preeclampsia. Application of Hydralazine 5 mg (i.v.) under close blood pressure monitoring was performed. Under spontaneous progression of labor, one head of the yet unknown conjoined twin was born. The patient developed eclamptic fits. Ceasing of seizures was achieved after implementing the loading dose of the MgSO_4_ protocol. A vaginal examination led to the unexpected diagnosis of conjoined twins. An emergency cesarean section under general anesthesia via a longitudinal midline incision was performed immediately. The born head was repositioned vaginally. The stillborn conjoined twins presented a female thoracopagus type that seemed to involve the heart. After 8 weeks, the woman was clinically fully recovered.

## 1. Introduction

Conjoined twins represent one of the rarest forms of twin gestation. They are always identical and occur in about 1 in every 200 sets of monozygotic twin pregnancies. The estimated overall incidence ranges from 1 in 50,000 to 1 in 250,000 live births [1–3]. However, about 40–60% of the cases are stillborn [2, 3].

Conjoined twins are suggested to result from aberrant embryogenesis in monozygotic twins. Two main theories are being proposed. In the fission theory, it is speculated that the origin of conjoined twins is an incomplete division of a single zygote at the primitive streak stage of the embryonic plate (15–17 days) [4]. In the fusion theory, it is proposed that a fertilized egg completely separates; however, stem cells lead to the fusion of both embryos [4, 5]. Importantly* high maternal and fetal risks* are present in all these conjoined twin cases, and even under the best of circumstances good outcomes for mother and both babies are rarely achieved. Considering termination of pregnancy might be the ultimate choice [6–8].

Preeclampsia is a pregnancy-associated disorder characterized by high blood pressure and significant proteinuria after 20 weeks of gestation. Eclampsia is a hypertensive disorder in pregnancy combined with convulsions [9].

Among the risk factors for hypertensive disorders are preexistent hypertension, existence of antiphospholipid syndrome or other coagulopathies, occurrence of certain biochemical markers such as the soluble fms-like tyrosine kinase-1 (sFlt-1)/placental growth factor (PLGF) ratio, status of primigravida, past obstetric history of preeclampsia, twin pregnancy, ethnicity, and low socioeconomic status.

The incidence of preeclampsia and related hypertensive disorders of pregnancy ranges from 2 to 5% for the United States, Canada, and Western Europe [10, 11], whereas in so-called developing countries, hypertensive disorders of pregnancy have the huge impact of 4–18% of all deliveries [10, 11]. The variation in incidence rates is due to the diversity of definitions, tests, and their methodologies and the differences in healthcare standards in the various African countries [10]. In fact, severe preeclampsia and eclampsia remain a significant public health threat in both developed and developing countries. They are accountable for 12% of all maternal deaths worldwide, which represent the third leading cause of maternal mortality and equal 76,000 women who die in childbirth every year [12]. 99% of all maternal deaths occur in so-called developing countries [13].

Apart from maternal mortality, hypertensive disorders in pregnancy have considerable adverse impacts on maternal, fetal, and neonatal health.

Early diagnosis, close prenatal management, and the choice of proper route of delivery will determine the best possible outcomes in both pathologies, respectively. In the present article, we are reporting a case of thoracopagus that was undiagnosed prior to delivery and combined with eclampsia and obstructed labor in Freetown, Sierra Leone, a low-resource country in sub-Saharan Africa.

## 2. Case Presentation

We report the case of a 27-year-old pregnant woman, gravida 3 para 2, who was presented to the maternity emergency unit of Princess Christian Maternity Hospital (PCMH) in Freetown at term in labor. The patient was referred from a health center for prolonged labor and arrest of descent at complete cervical dilatation despite being in active labor for the past 9 hours. She was an illiterate street trader. In terms of past obstetric history, there was a spontaneous vaginal delivery (SVD) at term of a healthy live born boy and a fresh stillbirth at term for an unknown reason. During this present pregnancy, she did not make any antenatal care visit. Therefore, blood pressure levels during pregnancy were undocumented and no obstetric ultrasound examination was performed. Her last menstrual date was unknown. Other relevant risk factors, such as gestational diabetes and infectious diseases, were also not known or documented.

Upon admission, the patient was awake and orientated and presented the following vital signs: blood pressure of 180/120 mmHg and a protein value of 3+ on urine dipstick test. Clinical examination revealed anasarca and hyperreflexia and a gravid abdomen with assumed term pregnancy and assumed singleton. The cervix was fully dilated with dystocia of labor; the presentation was cephalic. On auscultation with Pinard stethoscope, fetal heartbeat was absent. Ultrasound was not available. The findings led to the admission diagnosis of “obstructed labor with intrauterine fetal death (IUFD) and preeclampsia.”

The patient was admitted to the eclamptic ward to be stabilized, and application of Hydralazine 5 mg (i.v.) every 20 minutes under close blood pressure monitoring was performed.

Under spontaneous progression of labor (no application of oxytocin), the patient was transferred to the labor ward, where one head of the yet unknown conjoined twin was born. Even though blood pressure was controlled (140/95 mmHg), the patient developed eclamptic fits. Ceasing of seizures was achieved after implementing the loading dose of the MgSO_4_ protocol [14].

**Figure 1:**
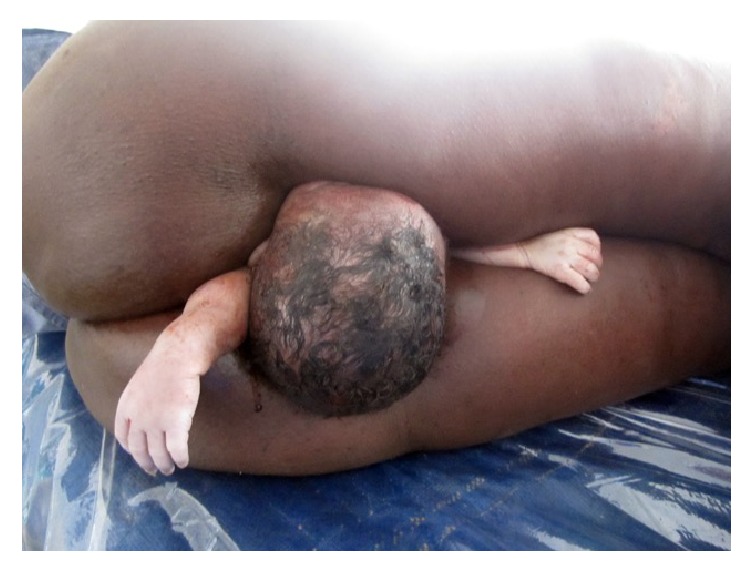
One head and two arms born.

The consultant obstetrician was now involved, and a new clinical assessment revealed a somnolent patient that was now stable, reflexes were low, and the blood pressure was controlled: 140/95 mmHg. The fundal height corresponded to term; there were no adequate contractions, and the fetal heart was absent. On vaginal examination, the presentation was left occiput posterior with stuck fetal head and a turtleneck phenomenon. With regard to the unknown presence of conjoined twins, the diagnosis of obstructed labor (suspicion of shoulder dystocia) and IUFD was confirmed and eclampsia was added. In an attempt to deliver the shoulders, manual extraction of both arms was performed as follows: reaching up along the dorsal shoulder blade, sweeping the humerus down, and thereby bringing the left arm out of the vagina. The same procedure was performed at the anterior shoulder for the right arm (Figure 1). Since shoulders were still not following and due to the unordinary presentation of the fetus, a second deep vaginal examination along the fetus' back was done, and further membranes were discovered. This led to the sudden and unexpected diagnosis of conjoined twins.

**Figure 2:**
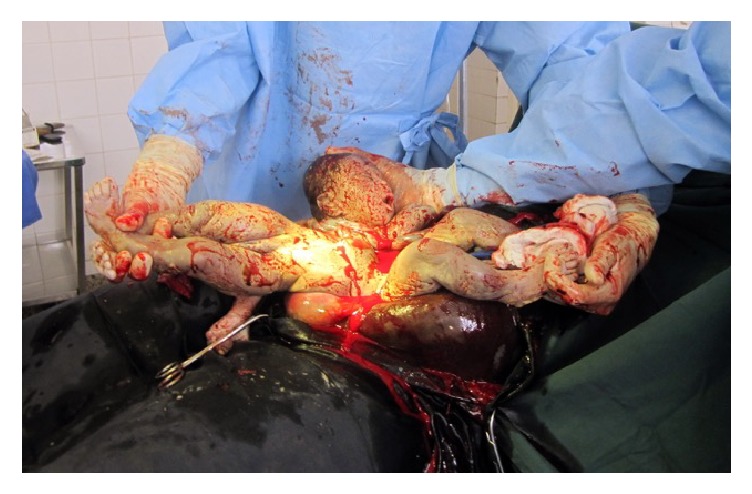
Surgery site during cesarean section.

**Figure 3:**
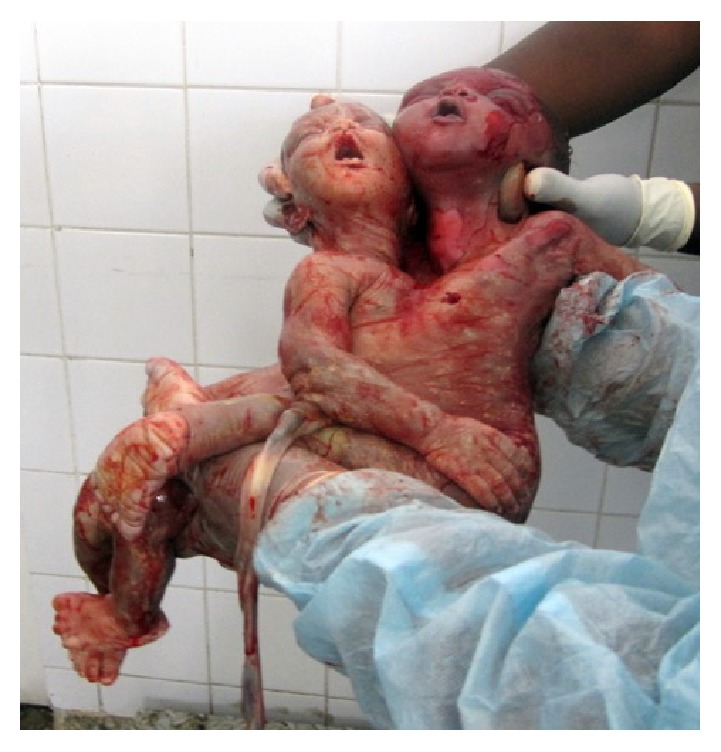
Stillborn thoracopagus-type conjoined twins.

An emergency cesarean section under general anesthesia via a longitudinal midline incision was performed immediately. The fetus was extracted by breech, whilst the born head was repositioned vaginally by a midwife (Figure 2). External inspection of the conjoined twins after surgery revealed a female thoracopagus type that seemed to involve the heart (Figure 3). Apgar score (Appearance, Pulse, Grimace, Activity, and Respiration) was 0/0/0, and pH was generally not available. The gestational age was estimated to be about 37–39 weeks of gestation.

The MgSO_4_ protocol was maintained further for 48 hours, and the antihypertensive therapy was continued with Hydralazine and later changed to 3 × 20 mg Nifedipine orally. The patient was kept under close monitoring of blood pressure, reflexes, respiration rate, and input-output evaluation.

During the postpartum period, there was no reappearance of seizures, and the anasarca disappeared fully. In the absence of proper laboratory facilities, no parameters concerning the hepatic or renal status could be done. Oral antihypertensive therapy was maintained for 6 weeks. On the control visit after 8 weeks, the patient was clinically fully recovered.

## 3. Discussion

Conjoined twins represent one of the rarest forms of twin gestation.

In the present article, we present the unique, and so far undescribed, case of a triple coincidence of conjoined twins, eclampsia, and obstructed labor. This scenario is further complicated due to unavailability of obstetric ultrasound in a low-resource setting.

### 3.1. The Challenge of Conjoined Twins

Anatomically, conjoined twins are classified based upon the site of attachment: thorax (thoracopagus), abdomen (omphalopagus), sacrum (pygopagus), pelvis (ischiopagus), skull (cephalopagus), and back (rachipagus). The extent of organ sharing, especially of the heart, determines the possibility and prognosis of a surgical separation procedure [2]. The most common types are thoracopagus [6, 15] with fusion from the anterior thorax to the umbilicus. A common pericardial sac is present in 90% of thoracopagus twins, and conjoined hearts are seen in 75% [16].

In general, there is higher predisposition towards female than male gender with a ratio of 3 : 1 [2, 3].

Early diagnosis of conjoined twins via ultrasound is reported in the first trimester but not before the 10th week of gestation [17]. Once the diagnosis is made, further characterization of the type and severity of the abnormality can be performed by three-dimensional ultrasound, computed tomography, or magnetic resonance imaging [18]. Based on these imaging techniques, the decision of carrying on with the pregnancy or its termination can be made.

Thus, early diagnosis, close prenatal management, and the choice of a proper route of delivery will assure the best possible outcome for mother and both babies [6–8, 19].

Nevertheless, the situation of conjoined twins carries high maternal and fetal risks. Even in high-resource settings, conjoined conditions present an enormous challenge of a catastrophic obstetric event. Even under optimal clinical care, a good outcome is rarely achieved [6]. Approximately 40–60% of conjoined twins arrive stillborn, and about 35% survive for only one day. The overall survival rate of conjoined twins is estimated between 5 and 25% [2, 3]. The surgical separation of conjoined twins is a delicate and risky procedure. Mortality rates for twins who undergo separation vary, depending on their type of connection and the organs they share. For example, twins joined at the sacrum, the base of the spine, have a 68% chance of successful separation, whereas in cases of twins with conjoined hearts at the left ventricular level, there are no known survivors. Although success rates have improved over the years, surgical separation is still rare. Since 1950, in about 75% of the cases, at least one twin has survived separation. After separation, most twins need intensive rehabilitation because of the malformation and position of their spines; they often have difficulties bending their backs and sitting up straight.

In West Africa between 1963 and 1978, 12 cases of conjoined twins have been reported [20]: 8 sets were live born and 4 sets were stillborn. The 8 live born sets were surgically separated either in local hospitals or abroad. Surgical separation was successful in 6 cases (in 2 cases both twins did not survive surgery). In 4 cases, one twin died during surgery or was sacrificed, whereas the other twin survived. In 2 cases, both twins initially survived surgery, but in one set both died about a month later. The most common type and the ones most likely to be live born were the omphalopagus twins [20].

In all of Africa, only two sets of nonseparated conjoined twins have ever been reported in the current literature to be alive, namely, (i) the 19-year-old female thoracopagus Maria and Consolata Mwakikuti, born and raised in Tanzania, where they graduated from high school in 2017 and aim to become teachers [21, 22], and (ii) the Ethiopian mother who delivered in May 2017 a set of live born male thoracopagus conjoined twins who seem to be sharing a common heart and lung [23].

Taking all this information into account, and with respect to the ethical controversy of pregnancy termination, it becomes clear in the case presented here that the early diagnosis via ultrasound would have been fundamentally important: a potential pregnancy termination could have avoided unnecessary but life-threatening maternal complications. The low-resource setting would not have allowed surgical separation, the overall risk for stillbirth in conjoined twins is significantly high, and even if the diagnosis had been made early and an elective cesarean section had been performed, the risk for unseparated twins to die soon after birth would have been very high. In contrast, the choice of pregnancy termination would have spared the mother undergoing unnecessary surgery and the experience of life-threatening complications of preeclampsia and eclampsia.

### 3.2. The Challenge of Preeclampsia and Eclampsia

Hypertensive disorders in pregnancy are one of the major causes of maternal mortality and morbidity worldwide [11, 24–26]. 12% of all maternal deaths are due to preeclampsia/eclampsia and HELLP syndrome (hemolysis, elevated liver enzymes, low platelets, and pain), representing the third leading cause of maternal mortality worldwide [12, 13]. Thus, the impact of the disease is felt more severely especially in low-resource countries, such as sub-Saharan African countries, where severe forms of preeclampsia and eclampsia are far more common [27–31].

In our case, the patient presented with several independently known risk factors for developing a hypertensive disorder in pregnancy: (a) unfavourable obstetric history, (b) twin pregnancy, (c) no visits to the antenatal care (ANC) clinic/late referral to the clinic, (d) ethnicity, and (e) low socioeconomic status.

#### 3.2.1. Unfavourable Obstetric History

The stillbirth of unknown origin in our patient's past obstetric history may allude to a history of preeclampsia in the previous pregnancy, since preeclampsia can lead to complications like placental abruption and IUFD. If she has had preeclampsia in a prior pregnancy, that would be a risk factor for the current pregnancy to get preeclampsia again.

#### 3.2.2. Twin Pregnancy

The incidence of preeclampsia in twin pregnancies is 2-3 times higher than that in singleton pregnancies [32]. Since the pathogenesis of preeclampsia is to be found in abnormal placentation, it is supposed that the higher incidence in twin pregnancies is due to the increased placental mass compared to singleton pregnancies.

Circulating biomarkers such as PAPP-A, PlGF, and sFlt-1 have been identified to play a role in diagnosis and prediction of preeclampsia [33, 34].

Similarly in twin pregnancies, circulating sFlt-1 levels and sFlt-1/PlGF ratios can be found twice as high as those in singleton pregnancies [35].

Among twin pregnancies, monochorionic types have the highest risk of preeclampsia [36].

#### 3.2.3. No Visits to ANC Clinic/Late Referral to the Clinic

In developed countries, pregnant women are commonly followed up by a healthcare specialist (doctor, midwife, or nurse) with frequent antenatal evaluations, even without presenting any risk factor. The role of early diagnosis and management of pregnancy-associated conditions has been numerously published worldwide. Special emphasis is placed on the importance of the first-trimester screening via ultrasound [37, 38]. Combined with clinical examination and detection of biochemical markers, it leads to early diagnosis and treatment of pregnancy-associated conditions and permits significantly reducing complications [33, 34, 39, 40]. Preeclampsia should be detected and appropriately managed before the onset of convulsions (eclampsia) and other life-threatening complications. Administering drugs for preeclampsia, such as magnesium sulfate, can lower a woman's risk of developing eclampsia.

Being exposed during her entire pregnancy to an undiagnosed preeclampsia and even having to undergo an eclamptic fit put the mother presented here under a life-threatening maternal mortality risk [25, 26, 30]. Moreover, we cannot even estimate whether she remains with residual kidney, liver, or brain damage, since laboratory parameters could not be performed.

The impact can clearly be seen in our case, where no visits to the ANC clinic and delay in the referral lead to undetected preeclampsia with the consequence of avoidable and life-threatening complication of eclampsia. Due to unavailability of early obstetric ultrasound, the conjoined twins still might not have been detected, even if the patient had attended ANC clinic.

### 3.3. The Challenge of a Low-Resource Setting

#### 3.3.1. The Presented Patient Case

The impact of an early diagnosis of pregnancy-associated conditions may be even greater in so-called developing countries, where significant avoidable maternal and neonatal morbidity and mortality often result.

This is perfectly reflected in our presented case. There is no doubt that access to healthcare is the main complicating factor for our patient.

It is well known that in developing countries medical conditions during pregnancy commonly advance to more complicated stages of disease, and many unreported births and deaths occur at home. Also, medical interventions may be ineffective due to late presentation of the cases.

1. Unavailability of early ultrasound led to undiagnosed conjoined twins. If known, termination could have been offered, or at least an elective cesarean section could have been performed, and the complication of obstructed labor would have been prevented.
2. No visits to ANC clinic led to undiagnosed preeclampsia and therefore to a more advanced stage and then finally to the unnecessary and dangerous complication of eclampsia.
3. Delay in referral and treatment led to obstructed labor and eclampsia.
4. Deficiency in skills leads to delay and complications of obstructed labor and eclampsia.
5. There is no doubt that the indication for cesarean section was already given on admission for obstructed labor and severe preeclampsia, respectively. Also the situation of severe preeclampsia in labor would have indicated an immediate onset of the MgSO_4_ protocol. The explanation can only be insufficiently skilled staff or insufficiently motivated staff.
6. Even to prevent a cesarean section would have been beneficial for the mother: being a postcesarean case with an uterine scar, the mother runs a high risk of further complications and even maternal death in a future childbirth when being in a low-resource setting.

Inaccessibility is a major barrier resulting in morbidity and mortality, which would otherwise have been prevented.

#### 3.3.2. The Setting of the Princess Christian Maternity Hospital (PCMH) in Sierra Leone

Sierra Leone is on the West Coast of sub-Saharan Africa. The population is about 7,09 million people and life expectancy is as low as 50,1 years for both sexes. The literacy rate is low with an average of 43%. Half of the population live on only 1,25 Dollar per day [41]. Many births occur at home, which leads to the fact that skilled health personnel are only present in 59,7% of all births in total. Furthermore, physicians density and nursing and midwifery personnel density are extremely low, 0,03/1000 and 0,8/1000, respectively, and are insufficient for the need of the population [41].

In April 2010, the government of Sierra Leone launched free healthcare services for pregnant women, lactating mothers, and children under 5. Nevertheless, maternal mortality is estimated at 1,360/100,000 live born births, and it is considered as one of the highest worldwide (also in the subregion) [42].

The Princess Christian Maternity Hospital (PCMH) is the biggest referral hospital for Obstetrics and Gynecology in this country with about 4,000 births per year.

Laboratory investigations are limited to hemoglobin (Hb), blood grouping, malaria, human immunodeficiency virus (HIV), and urine tests using dip sticks; a limited blood bank facility is available. Very often there is a lack of basic equipment and medication, such as IV lines, surgical sutures, antibiotics, antihypertensive medication, MgSO_4_, Ringer, or N/S, due to a poor distribution system for drugs. Unstable power supply leads to inaccessibility to electronic devices, such as perfusors or cardiotocogram (CTG). Concerning ultrasound, there is one machine with only an abdominal probe and no Doppler available. The machine is not accessible in the emergency department, labor ward, or theatre; and ultrasound skills are possessed by only one consultant obstetrician. The number of trained health professionals is unacceptably inadequate. Skills and motivation of health workers, that is, midwives, nurses, and doctors, are insufficient with regard to the medical challenges faced. This leads to a lack of implementation of mandatory guidelines and structured procedures; thus timelines are not respected, often through incapacity or even neglect.

#### 3.3.3. The Low-Resource Setting in a Global Context

In the year 2000, the international community committed to 8 Millennium Development Goals (MDG) to be achieved by 2015 [43]. MDG number 5 commits to reducing maternal mortality by three quarters worldwide, which would have required an annual decline of 5.5% until 2015. However, between 1990 and 2013, the global maternal mortality ratio declined by only 2.6% per year.

Albeit shocking, the course of events in the case presented here is more common than unusual—seen from a global perspective and especially in the context of a low-resource setting [10, 13, 24, 25, 27, 29, 31, 43]. It is well known that in developing countries medical conditions during pregnancy commonly advance to more complicated stages of disease, and many unreported births occur at home. Also, medical interventions may be ineffective due to late presentation of the cases [25, 26]. Every year, over half a million women die globally from pregnancy and childbirth-related complications [13]. Out of these, 99% of all maternal deaths occur in so-called developing countries.

The main causes for maternal deaths accounting for 80% of the cases are severe bleeding, infections, preeclampsia and eclampsia, and unsafe abortion [12, 13].

Most maternal deaths are avoidable, since the healthcare solutions to prevent or manage complications are well known. But why do not women get the care they need?

According to World Health Organization (WHO), worldwide factors preventing women from receiving or seeking care during pregnancy and childbirth are poverty, distance, lack of information, cultural practices, and inadequate services [13].

Access to ANC clinic, facility deliveries, a skilled birth attendant at delivery, and family-planning methods are crucial in preventing these complications. Timely management and treatment can make the difference between life and death.

The higher number of maternal deaths in sub-Saharan Africa reflects inequities regarding access to health services.

“Access” in this case does not only mean rural and remote areas but also stands for

- the gap between rich and poor,
- lower social status of women; lack of education leads to not claiming health service that is provided,
- traditional health practices which are usually inadequate to detect medical conditions early,
- low numbers of skilled health workers.

World Health Organization reports that only 46% of women in low-income countries benefit from skilled care during childbirth [12, 13]. This means that millions of births are not assisted by a midwife, a doctor, or a trained nurse. Many unreported births and deaths occur at home.

Moreover, women in developing countries have on average many more pregnancies than women in developed countries, and their lifetime risk of death due to pregnancy is thus significantly higher.

## 4. Summary

In the present article, we present the unique and so far undescribed case of a triple coincidence of conjoined twins, eclampsia, and obstructed labor which occurred in Freetown, Sierra Leone, a low-resource country in sub-Saharan Africa.

Conjoined twins represent one of the rarest forms of twin gestation. The situation of conjoined twins carries high maternal and fetal risks, thus holding the absolute challenge of preventing a catastrophic obstetric event. Even under the best of circumstances, a good outcome is rarely achieved. Therefore, termination of pregnancy may be the choice. Although success rates have improved, surgical separation of conjoined twins is a delicate and risky procedure. Cases where at least one twin has survived separation are reported to be about 75%. In Africa, there are only two sets of nonseparated thoracopagus conjoined twins reported to be alive: the 19-year-old Maria and Consolata Mwakikuti from Tanzania and the male thoracopagus set born in May 2017 in Ethiopia [21–23].

Severe preeclampsia and eclampsia have remained a significant public health threat in both developed and developing countries, as they have considerable adverse impacts on maternal, fetal, and neonatal health. However, the impact of the disease is felt more severely, especially in low-resource countries, where conditions are often complicated, since medical interventions may be ineffective due to late presentation of cases. Advances to more complicated stages of disease are common.

The role of early diagnosis for conjoined twins and preeclampsia, respectively, is crucial.

The occurrence of both conditions, conjoined twins and preeclampsia, in a developing country is demonstrated in the present article to be the main complicating factor for the fetal and maternal outcomes (fetal mortality and maternal morbidity).

Worldwide factors preventing pregnant women from seeking or receiving healthcare, such as poverty, distance, lack of information, inadequate services, and cultural practices, are discussed.

In the 21st century, it is unacceptable that mothers die from preventable conditions or go through life-threatening preventable complications.

If substantial reductions in maternal mortality are to be achieved, universal coverage of life-saving interventions needs to be matched with comprehensive emergency care and overall improvements in the quality of maternal healthcare.

To improve maternal health worldwide, barriers that limit access to quality maternal health services must be identified and addressed at all levels of the health system.

The implementation of guidelines and policies and the application of a profound quality management system would crucially improve performances at health centers and hospitals in low-resource settings and therefore lead to a better functional medical system in its entirety.
